# An investigation of scattered light integrating collector technology for rapid blood culture sensitivity testing

**DOI:** 10.1099/jmm.0.001896

**Published:** 2024-10-03

**Authors:** L. White, R. Hammond, R. J. Shorten, J. P. Derrick

**Affiliations:** 1 Department of Microbiology, Lancashire Teaching Hospitals NHS Foundation Trust, England, UK; 2 Infection and Global Health Division, School of Medicine, University of St Andrews, St Andrews, UK; 3 Honorary Senior Lecturer, University of Manchester, Manchester, UK; 4 School of Biological Sciences, University of Manchester, Manchester, UK

**Keywords:** antibiotic, blood culture, Gram-negative, sensitivity, SLIC, susceptibility

## Abstract

**Introduction.** Sepsis rates are increasing, with Gram-negative organisms representing a large proportion of bloodstream infections. Rapid antibiotic administration, alongside diagnostic investigations, is required for the effective management of these patients.

**Gap statement.** Current diagnostics take ~48 h for a final report; therefore, rapid diagnostics are required.

**Aim.** This study investigated a novel antibiotic sensitivity method, the scattered light integrating collector (SLIC), combined with a rapid identification method using matrix-assisted laser desorption/ionization time of flight (MALDI-TOF) technology to determine if an accurate identification and susceptibility result can be provided within 4 h of a positive blood culture report.

**Methodology.** A total of 47 blood cultures containing Gram-negative bacteria from 46 patients were processed using the MALDI-TOF Biotyper Sepsityper for identification directly from the blood and the SLIC instrument for susceptibility testing. All organisms were also tested using the current standard workflow used in the host laboratory. Categorical agreement (CA), major errors (MaEs) and very major errors (VMEs) were determined.

**Results.** SLIC produced susceptibility results with a 71.9% CA, 30.6% MaE and 17.5% VME. The median difference in time to the final result was 44.14 (43 : 05–45 : 15) h earlier compared to the current method.

**Conclusion.** We conclude that SLIC was unable to consistently provide sufficiently accurate antibiotic susceptibility results compared to the current standard method.

## Introduction

Sepsis is a life-threatening condition that affects ~245 000 people in the UK every year, with a mortality of ~20% [1]. Bacteraemia is a common reason for sepsis, and the incidence of confirmed bloodstream infections (BSIs) has increased by 10.8% since 2017 [2]. Gram-negative organisms are frequently isolated from the bloodstream, with *Escherichia coli *being the most common. Infection with *E. coli* has increased from 60.4/100 000 population in April 2012/2013 to 67.1 in 2021/2022, indicating that bacteraemia and sepsis are increasing [3].

Rapid administration of empiric antibiotics is required in septic patients to combat an increased risk of mortality and morbidity [4,8]. Targeted therapy using results from culture reduces the risk of toxic side effects from broad-spectrum antibiotics, helps to reduce the impact on the patients’ normal bacterial microbiota and prevents the spread of antimicrobial resistance (AMR) [8,10]. The group of bacteria to which *E. coli* belongs (Enterobacterales) are currently the major reservoir for AMR BSIs and account for 80.3% of all AMR BSIs. It is therefore important to ensure accurate identification and sensitivity testing of organisms causing sepsis. Such a policy will lead to a reduction in the use of broad-spectrum antibiotics and further retard the development of AMR [2].

Current diagnostic tests for bacteraemia using conventional culture and susceptibility techniques can take 48 h. There is a clear imperative to reduce the time from specimen receipt to result so that antimicrobial therapy can be optimized. More rapid phenotypic [11,17] and genotypic methods [18,20] have been proposed.

In this study, we set out to evaluate the utility of the scattered light integrating collector (SLIC) as a rapid automated sensitivity testing device for BSIs in a clinical laboratory setting. The SLIC instrument uses static laser light scattering to determine the phenotypic susceptibility of a cultured bacterium to antibiotics within 4 h. A laser is directed through a sample that reflects, refracts and diffuses, depending on if the sample has scattered or absorbed the photons. If there is growth of the organism, there will be increased scattering of photons. A photodiode measures the scattered photons and reads the signal as the change in an electric current, which is recorded over time. A suspension of bacteria scatters the light based on the number and morphology of the cells within the suspension [21].

Here, we examine the performance of SLIC in determining antimicrobial susceptibility profiles of Enterobacterales from positive blood cultures compared to conventional methods in a clinical laboratory setting. Time to the availability of the result, as well as any hypothetical antimicrobial management changes, was also examined.

## Methods

**Fig. 1.**
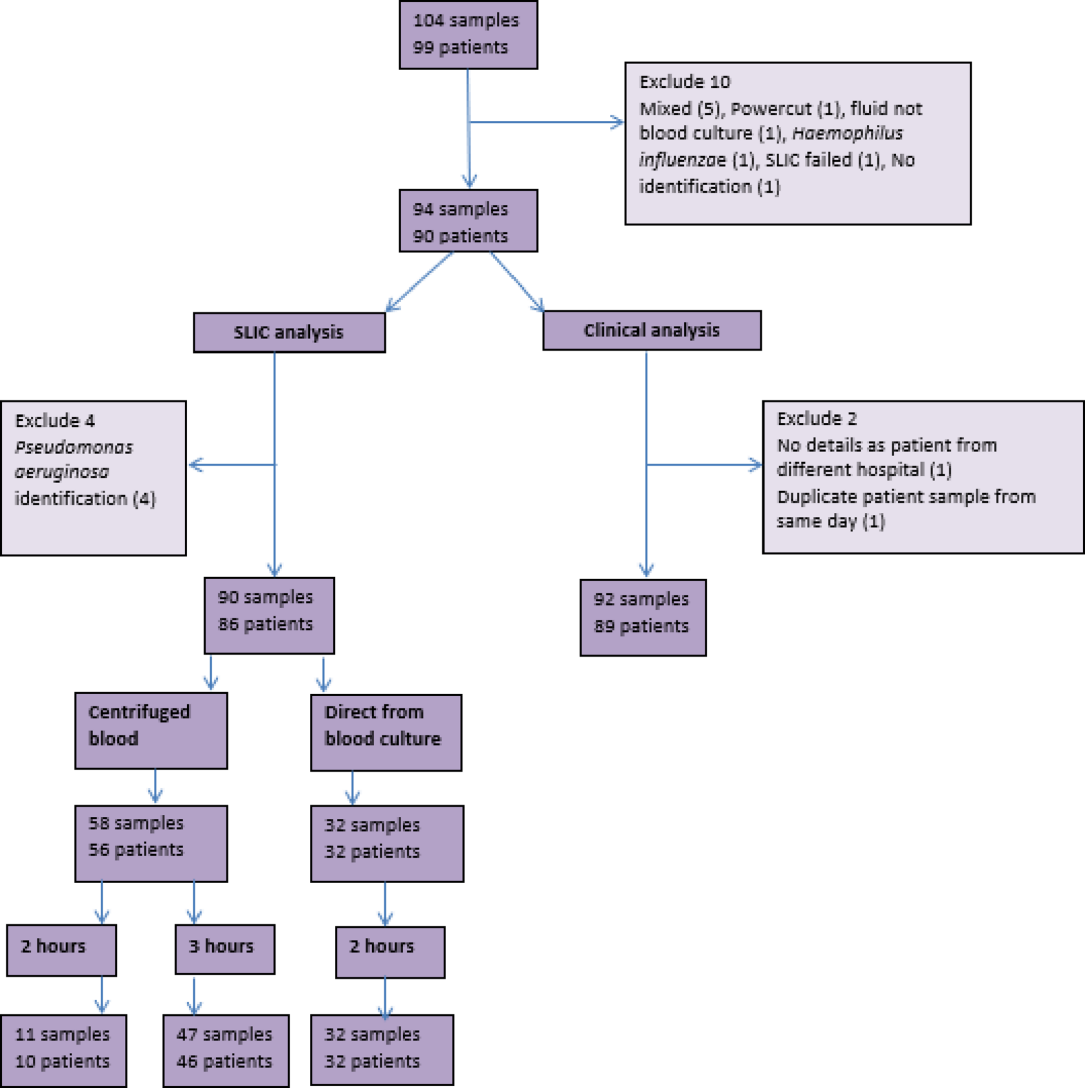
Breakdown of samples for SLIC and clinical analysis.

This comparative diagnostic study was carried out in Lancashire Teaching Hospitals NHS Foundation Trust, a secondary care hospital in the North-West of England with ~900 beds across two sites, between 9 June 2021 and 17 December 2021. Forty-seven positive blood culture samples from 46 patients, which were flagged positive by the BacT/ALERT system and contained Gram-negative bacilli on microscopy on the study days, were included and processed using the current laboratory method as well as the study method (SLIC). Exclusions included any non-Enterobacterales isolates and those that had their SLIC testing interrupted (Fig. 1).

### Current laboratory method

**Fig. 2.**
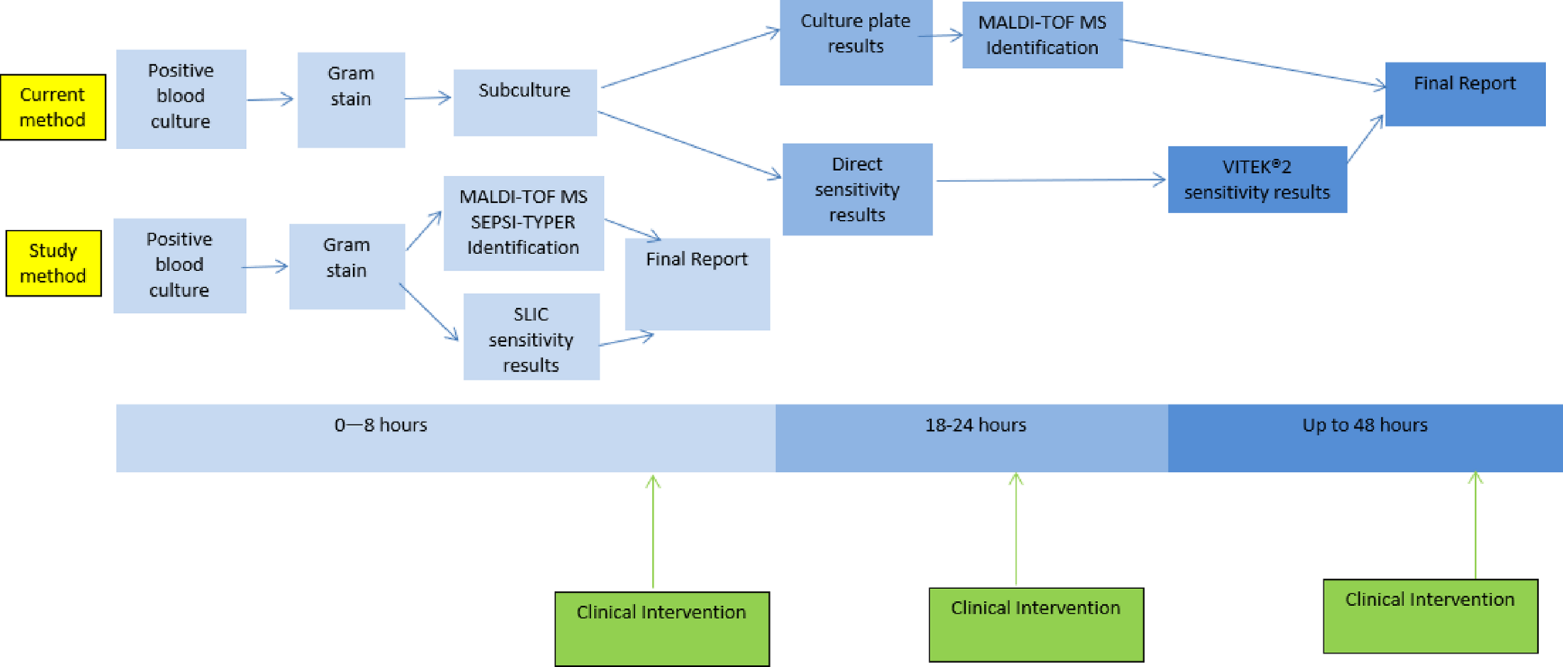
The current laboratory positive blood culture workflow for Lancashire Teaching Hospitals NHS Foundation Trust using the direct sensitivities, MALDI-TOF MS for identification and the VITEK®2 (with pivmecillinam and trimethoprim using EUCAST disc diffusion) for sensitivity testing as well as the study method workflow.

Blood cultures were incubated in the BacT/ALERT® 3D Microbial Detection System (bioMérieux). Positive cultures were processed as presented in the workflow in Fig. 2, with Gram stain, direct antimicrobial susceptibility via disc diffusion [European Committee on Antimicrobial Testing (EUCAST)] and sub-culture for identification the following day using mass spectrometry (MALDI Biotyper®, Bruker). Definitive susceptibility results were obtained using a VITEK®2 (bioMérieux) instrument.

### SLIC

Organisms from positive blood culture bottles were identified directly from liquid culture using a matrix-assisted laser desorption/ionization time of flight (MALDI-TOF) Sepsityper, according to the manufacturer’s recommendations and as previously described [22]. Liquid media from positive blood culture bottles were centrifuged at 1500 ***g*** for 4 min to remove inhibiting substances, prior to the supernatant being diluted 1 : 10 in sterile PBS. This sample was then added to 880 µl of media to make a 1 : 500 dilution in semi-micro-optical cuvettes, with the final antibiotic concentrations (EUCAST resistant breakpoint MIC), as detailed in Table 1 [23]. Positive (patient sample and media only) and negative (uninoculated media) controls were also included. Sensitivity testing was carried out on SLIC for 3 h, at which point output data were produced.

**Table 1.** Final concentrations of antibiotics in the SLIC analysis

Antibiotic	Final cuvette solution concentration (µg ml^−1^)
Amoxicillin	8
Pivmecillinam	8
Co-amoxiclav	8
Piperacillin–tazobactam	8
Cefuroxime	8
Ceftazidime	4
Ceftriaxone	2
Cefpodoxime	1
Cefoxitin	8
Ertapenem	0.5
Meropenem	8
Gentamicin	2
Ciprofloxacin	0.5
Trimethoprim	4

### Data analysis

The data produced by the SLIC instrument were run through a specifically designed macro. This determined the sensitivity of an organism to an antibiotic when there was a 50% reduction in its growth compared to the positive control. The identification results of the study method were compared with the current laboratory method, and the percentage identification match was recorded. The sensitivity testing of the study method using SLIC was compared with the chosen ‘gold standard’ (VITEK®2 for all antimicrobials except pivmecillinam and trimethoprim, which were tested by EUCAST disc diffusion). The SLIC susceptibility result was assessed for categorical agreement (CA), in which the percentage of isolates providing concordant results from SLIC and the current method was calculated. Major errors (MaEs) were defined as a resistant result by SLIC and a sensitive method by the current method. Very major errors (VMEs) were defined as a sensitive result by SLIC and a resistant result by the current method [24,28].

### Clinical results

A clinical microbiologist retrospectively accessed the prescribing data for each patient and assessed if the patient was on appropriate antibiotics at the time of the positive blood culture. The time taken to the final identification and susceptibility report was calculated from the time the Gram stain was reported to the clinical teams. These results were assessed for normality using Kolmogorov–Smirnov and Shapiro–Wilk tests in SPSS® and were found to be not normally distributed. A Wilcoxon-Signed Rank-Sum test was therefore used to assess for a statistically significant difference in time taken to the final report between the existing method and the SLIC study method. The null hypothesis was that the difference in time taken to the final report was zero.

## Results

### Identification

Forty-seven samples from 46 patients were processed using the current laboratory method and the study SLIC method. Of these, *E. coli* was isolated the most often (57.4%), followed by *Klebsiella pneumoniae* (8.5%) and *Proteus mirabilis* (8.5%), with other Enterobacterales making up the remainder. There was a 100% agreement in identification between the study method and the current method.

### Antibiotic susceptibility testing

Overall, the CA between the current laboratory method and SLIC was 71.9%. There was no antibiotic that produced 100% concordance, although gentamicin and amoxicillin performed at 93.6 and 90%, respectively (Table 2). Piperacillin–tazobactam performed the worst, providing a concordance of 17.0%, whilst the remainder of the antibiotics tested were above 50%. The median number of MaE was 8 (Inter Quartile Range [IQR]: 5–13), with all antibiotics having at least one MaE reported. Amoxicillin had the fewest, with 2, and piperacillin–tazobactam had the most, at 38. The median number of VME was 1 (IQR: 0–2), and no VMEs were reported for four antibiotics (ceftazidime, ertapenem, meropenem and gentamicin), with six for cefoxitin as the highest number. In total, out of 604 tests, there were 150 MaEs (30.6%) and 20 VMEs (17.5%).

**Table 2.** Data and categorical agreement from the SLIC method compared with current laboratory methods for each antibiotic

Antibiotic*	**Categorical agreement**	Major errors (MaEs)†	Major error rate (%)	Very major errors (VMEs)‡	Very major error rate (%)
Amoxicillin	90.0% (36/40)	2	12.5	2	8.3
Pivmecillinam	72.3% (34/47)	10	25.0	3	42.9
Co-amoxiclav	53.2% (25/47)	21	77.8	1	5.0
Piperacillin–tazobactam	17.0% (8/47)	38	90.5	1	20.0
Cefuroxime	80.9% (38/47)	7	21.9	2	13.3
Ceftazidime	76.6% (36/47)	11	25.0	0	0.0
Cefpodoxime	70.2% (33/47)	13	33.3	1	12.5
Cefoxitin	78.7% (37/47)	4	11.1	6	54.5
Ertapenem	57.4% (27/47)	20	42.6	0	0.0
Meropenem	89.4% (42/47)	5	10.6	0	0.0
Gentamicin	93.6% (44/47)	3	6.8	0	0.0
Ciprofloxacin	78.7% (37/47)	8	19.5	2	33.3
Trimethoprim	78.7% (37/47)	8	22.9	2	16.7
**Total**	71.9% (434/604)	150	30.6	20	17.5

1*Ceftriaxone was the 14th antibiotic tested on SLIC; however, this is not tested on the routine laboratory panel, so it was excluded from this analysis. Amoxicillin has fewer total tests compared to the other antibiotics, as some organisms are not tested for amoxicillin due to intrinsic resistance (e.g., *Citrobacter* species,and *Enterobacter* species).

2†MaE rates are calculated using the total number of susceptible organisms.

3‡VMEs are calculated using the total number of resistant organisms.

### Timings

The median time taken for patients to be placed on effective antibiotics was 0 min (0–232 min) due to a large number of patients in the cohort (62) already being treated with effective antibiotics when the blood culture was reported as positive.

Using the current method in the laboratory, the median time (IQR) for the final identification and sensitivity result to be available was 47 h 48 min (46 : 38–48 : 45). In comparison, the study method found that, for the 47 samples tested for 3 h, the median time (IQR) was 3 h 35 min (3 : 30–3 : 40). When compared to the median time for the current method, using SLIC would allow for the final identification and sensitivity report to be available 44 : 14 h earlier (43 : 05–45 : 15) (*P*=1.4e-14), which therefore means that the null hypothesis that there is no difference in time taken to the final report between the two methods was rejected.

## Discussion

Overall, the performance of SLIC was not comparable to that of the current workflow, with a total CA for all the tested antibiotics of 71.9%. The study method did, however, produce a result within the 4-h objective and nearly 2 days earlier than the current standard method. This CA result would mean that this method does not meet the current criteria necessary for clinical implementation. This is because the CA for the introduction of a new susceptibility method should be 90% and above for acceptance [28,30]. The acceptable rates for MaE and VME vary within the literature: the VME rate depends on the number of resistant isolates tested, although the generally accepted values are <1.5% for VME and <3% for MaE [27,30].

The first and second most frequently isolated bacterial species in the study correspond to the current first and second most common Enterobacterales isolated from blood cultures in England (*E. coli* and *K. pneumoniae*), with *Proteus* species the third most common [2]. The 100% concordance with the Sepsityper MALDI method is in agreement with literature reports, which show >90% concordance with organism identification using blood directly from the positive blood culture bottle [31,34]. Identification of the pathogen can be useful for the management of patients, as some organisms are known to have specific resistance patterns. Given, however, that the most commonly isolated organisms were *E. coli*, *K. pneumoniae* and *P. mirabilis*, none of which are known to have intrinsic mechanisms conferring clinically significant resistance; having this identification result for these organisms does not help with any expected sensitivity results.

The SLIC instrument did not perform as well as expected compared to a previous study carried out on positive blood cultures by the Hammond Research Group (University of St Andrews), where an average CA of 95.5% and the rate of errors of 4.1% MaE and 7.1% VME for a 2-h analysis were demonstrated [35]. The previous study was conducted for 2 h, not 3, but, in this study, the CA was 71.9% and the MaE rate was 30.6%. The VME rate was 17.5% when the SLIC data were collected after 3 h. This reduction in CA compared to the previous study on SLIC is unexpected, although the study methods were not the same. The SLIC instrument provided for this study was a bespoke 16-well model, and an increased number and range of antibiotics were used. Both methods used only Enterobacterales, with the majority being *E. coli* in both studies. The differences in instrument, timing and antibiotic panel and the fact that this study tested more antibiotics may explain the difference in results produced. In this study, the identification of piperacillin–tazobactam resistance was responsible for the majority of errors; this was not tested in the previous study, which may explain some of the differences in results. In addition, although extensive validation studies had been carried out on the 6-well instrument, the 16-well instrument was the first of its kind and was only validated in its capacity to detect the growth of *E. coli* [American Type Culture Collection (ATCC) strain 25922] and *Staphylococcus aureus* (ATCC stain 25923) grown in brain heart infusions and Mueller Hinton broth, which may explain the disparity between the two instruments.

Of the antibiotics tested, the poorest performer was piperacillin–tazobactam, and the best was gentamicin. These observations are similar to a study using Alfred 60AST (Alifax), which found the most discrepant results for piperacillin–tazobactam and managed to correct some of these with modification of the drug formulation by the manufacturer [36]. The resulting variation dependent on the method is said to be a known problem for piperacillin–tazobactam, which may explain the poor performance on SLIC [37].

There were variable CA results across the different antibiotics tested. One potential reason might be attributable to the fact that true MIC results can be one dilution on either side of the reported result. This could change whether the result is reported as resistant or susceptible, which could affect a comparative study between the two methods. There could also be variation due to the antibiotic stability when the dilutions were being prepared, frozen and then thawed, which may vary between antibiotics. The data were reviewed to examine whether bacteriostatic or bacteriocidal categorization had an influence on the results, but this does not appear to be the case: 12 of the antibiotics were bacteriocidal, and trimethoprim was the only bacteriostatic drug.

This SLIC instrument did not perform favourably in comparison with the standard method, which, unfortunately, is not what has been seen in other rapid susceptibility testing platform analyses. Platforms such as QMAC-dRAST™ (based on microfluidics), Accelerate Pheno®, Specific Reveal™ and Alfred 60AST (Alifax) have shown better performance with CA of 90.6–97.9%, VME of 0.0–3.3%, MaE of 0.0–2.5% and ME of 0.7–7.4% within 7 h [12,14, 17, 36, 38,54].

In this study, the median time to effective antibiotics was zero, indicating that the patients were already on effective antibiotics in the majority of cases. Empiric antibiotics for sepsis are based on local epidemiology and are designed to cover the most common organisms in a specific clinical context: locally, this does not include a high number of carbapenem-resistant Enterobacterales. This reasoning does not consider if the antibiotics were appropriate or unnecessarily broad spectrum. This was also seen in other studies, where patients were already on effective antibiotics 75–94% of the time [42,55], although this is contrary to other studies that report lower numbers of patients already on appropriate antibiotic therapy. This may be related to the antibiotic resistance rates and choice of empiric treatment in the local study area [6,42, 56,58]. A high percentage of patients undergoing treatment on effective antibiotics does not necessarily support the case for rapid susceptibility tests—the patients are being treated for the infecting organism already. The availability of rapid susceptibility results, however, can allow for the use of narrow-spectrum antibiotics. This can potentially reduce side effects such as *Clostridium difficile* and drug toxicity, and improve time to oral switch, which can facilitate hospital discharge and reduce length of stay. In addition, having information to allow for a de-escalation of antibiotics helps with antimicrobial stewardship and development of resistance.

Rapid identification and sensitivity results can potentially benefit a patient clinically; however, a limiting factor that needs to be considered is whether the rapid results are going to be acted upon clinically once the testing is carried out. Many laboratories, including the one hosting this study, have specific opening hours of 8 am–6 pm on weekdays and 8–1 pm on weekends, and positive blood cultures are not processed outside of these hours. This may negate the benefit of the rapid positive result if it becomes available out of hours. This is a factor that would need to be taken into consideration if introducing a rapid method into the department.

There are several limitations to this study, including the fact that, although the VITEK®2 was noted as the ‘gold standard’ for the majority of the antibiotics tested in this study, it is not free from error itself. There may have been cases where the VITEK®2 result was incorrect and the SLIC result was correct because the VITEK®2 was not 100% accurate. VITEK®2 has previously been shown to have a minimum inhibitory concentration agreement (and therefore CA) of 84.2–95.6 %, a VME of 0.4% and MaE of 0.5% when compared to broth microdilution [59]. This was not investigated within this study, and the VITEK®2 results were used as the defined laboratory standard. Initially, discrepancies were going to be investigated further, but unfortunately, due to the high number, this was logistically not possible. In addition, there were only 47 samples tested for 3 h; additional results may help to determine the performance of this method. The study also did not look at mixed cultures, which can often happen within the laboratory or *Pseudomonas* spp. isolates: both would need to be assessed in the future. This study focussed on Enterobacterales—future studies should include all Gram-negative and Gram-positive organisms. A larger sample size, with a wider array of organisms, would allow the results to be generalized to all positive blood cultures; these results can only apply to *Enterobacterales*. Pre-analytical parameters were not assessed, including the time taken for the blood culture to get to the laboratory (this is being addressed in the current Standards for Microbiological Investigation). There was also an absence of carbapenem-resistant organisms and a low incidence of gentamicin resistance, which means that these antibiotics have not been tested thoroughly; the results for these antibiotics may therefore be skewed. Further study is required to provide additional information on the performance of SLIC, and improvements to the method can be made. The antibiotic solutions were created by diluting powders along with a freezing and thawing cycle, which could introduce inaccuracies. In order to overcome this, alternative methods, such as impregnated discs in the cuvettes or antibiotic-impregnated discs, could be used in the future. The samples were incubated for 3 h to gain the sensitivity results: an alternative option would be to incubate for longer to assess if this intervention changes the overall results.

## Conclusion

The system under investigation during this study (SLIC) has not performed sufficiently well to meet the standards necessary for clinical implementation at the present time without further investigation. However, the results do suggest that rapid susceptibility results could provide a final report significantly quicker than the current standard method, and this could potentially lead to more targeted antibiotic treatment, although this would need to be studied further.

## Abbreviations

AMR: antimicrobial resistance
ATCC: American Type Culture Collection
BSIs: bloodstream infections
CA: categorical agreement
EUCAST: European Committee on Antimicrobial Testing
HCRW: Health Research Agency and Health and Care Research Wales
IQR: inter quartile range
MaEs: major errors
MALDI-TOF MS: matrix assisted laser desorption/ionisation -time of flight mass spectrometry
MIC: minimum inhibitory concentration
PBS: phosphate buffered saline
SLIC: scattered light integrating collector
VMEs: very major errors

## References

[1] The UK Sepsis Trust (2022). The sepsis manual. https://sepsistrust.org/professional-resources/education-resources/.

[2] (2023). UK Health Security Agency. English surveillance programme for antimicrobial utilisation and resistance (ESPAUR) Report 2022 to 2023.

[3] (2024). UK Health Security Agency. Annual epidemiological commentary: Gram negative, MRSA, MSSA bacteraemia and *C. difficile* infections, up to and including financial year 2022 to 2023.

[4] Rhodes A, Evans LE, Alhazzani W, Levy MM, Antonelli M (2017). Surviving sepsis campaign: International Guidelines for management of sepsis and septic shock: 2016. Intensive Care Med.

[5] Kumar A, Ellis P, Arabi Y, Roberts D, Light B (2009). Initiation of inappropriate antimicrobial therapy results in a fivefold reduction of survival in human septic shock. Chest.

[6] Ibrahim EH, Sherman G, Ward S, Fraser VJ, Kollef MH (2000). The influence of inadequate antimicrobial treatment of bloodstream infections on patient outcomes in the ICU setting. Chest.

[7] Paul M, Shani V, Muchtar E, Kariv G, Robenshtok E (2010). Systematic review and meta-analysis of the efficacy of appropriate empiric antibiotic therapy for sepsis. Antimicrob Agents Chemother.

[8] Evans L, Rhodes A, Alhazzani W, Antonelli M, Coopersmith CM (2021). Surviving sepsis campaign: International Guidelines for Management of Sepsis and Septic Shock 2021. Crit Care Med.

[9] Masterton RG (2011). Antibiotic de-escalation. Crit Care Clin.

[10] Donner LM, Campbell WS, Lyden E, Van Schooneveld TC (2017). Assessment of rapid-blood-culture-identification result interpretation and antibiotic prescribing practices. J Clin Microbiol.

[11] (2022). European Committee on Antimicrobial Susceptibility Testing. EUCAST RAST Method’ Version 2.0.

[12] Choi J, Jeong HY, Lee GY, Han S, Jin B (2017). Direct, rapid antimicrobial susceptibility test from positive blood cultures based on microscopic imaging analysis. Sci Rep.

[13] Kim J-H, Kim TS, Jung HG, Kang CK, Jun K-I (2019). Prospective evaluation of a rapid antimicrobial susceptibility test (QMAC-dRAST) for selecting optimal targeted antibiotics in positive blood culture. J Antimicrob Chemother.

[14] Sánchez-Carrillo C, Pescador P, Ricote R, Fuentes J, Losada C (2019). Evaluation of the Alfred AST® system for rapid antimicrobial susceptibility testing directly from positive blood cultures. Eur J Clin Microbiol Infect Dis.

[15] Ehren K, Meißner A, Jazmati N, Wille J, Jung N (2019). Clinical impact of rapid species identification from positive blood cultures with same-day phenotypic antimicrobial susceptibility testing on the management and outcome of bloodstream infections. Clin Infect Dis.

[16] Göransson J, Sundqvist M, Ghaderi E, Lisby JG, Molin Y (2023). Performance of a system for rapid phenotypic antimicrobial susceptibility testing of Gram-negative bacteria directly from positive blood culture bottles. J Clin Microbiol.

[17] Tibbetts R, George S, Burwell R, Rajeev L, Rhodes PA (2022). Performance of the reveal rapid antibiotic susceptibility testing system on Gram-negative blood cultures at a large urban hospital. J Clin Microbiol.

[18] Berinson B, Both A, Berneking L, Christner M, Lütgehetmann M (2021). Usefulness of BioFire FilmArray BCID2 for blood culture processing in clinical practice. J Clin Microbiol.

[19] Krifors A, Rådberg G, Golbob S, Omar Z, Svensson C (2020). The clinical impact of implementing genmark eplex blood culture panels for around-the-clock blood culture identification; a prospective observational study. Infect Dis.

[20] Bard JD, Lee F (2018). Why can’t we just use PCR? The role of genotypic versus phenotypic testing for antimicrobial resistance testing. Clin Microbiol Newsl.

[21] Hammond RJH, Falconer K, Powell T, Bowness R, Gillespie SH (2022). A simple label-free method reveals bacterial growth dynamics and antibiotic action in real-time. Sci Rep.

[22] Ponderand L, Pavese P, Maubon D, Giraudon E, Girard T (2020). Evaluation of rapid Sepsityper® protocol and specific MBT-Sepsityper module (Bruker Daltonics) for the rapid diagnosis of bacteremia and fungemia by MALDI-TOF-MS. Ann Clin Microbiol Antimicrob.

[23] On EC, antimicrobial susceptibility testing. EUCAST clinical breakpoint tables V.11, valid from 2021-01-012021. https://www.eucast.org/ast_of_bacteria/previous_versions_of_documents.

[24] Edelmann A, Pietzcker T, Wellinghausen N (2007). Comparison of direct disk diffusion and standard microtitre broth dilution susceptibility testing of blood culture isolates. J Med Microbiol.

[25] Hombach M, Böttger EC, Roos M (2013). The critical influence of the intermediate category on interpretation errors in revised EUCAST and CLSI antimicrobial susceptibility testing guidelines. Clin Microbiol Infect.

[26] Jorgensen JH (1993). Selection criteria for an antimicrobial susceptibility testing system. J Clin Microbiol.

[27] Murray PR, Niles AC, Heeren RL (1987). Comparison of a highly automated 5-h susceptibility testing system, the Cobas-Bact, with two reference methods: Kirby-Bauer disk diffusion and broth microdilution. J Clin Microbiol.

[28] US Food and Drug Administration (2003). Class II special controls guidance document: antimicrobial susceptibility test (AST) systems; guidance for industry and FDA.

[29] Reller LB, Weinstein M, Jorgensen JH, Ferraro MJ (2009). Antimicrobial susceptibility testing: a review of general principles and contemporary practices. Clin Infect Dis.

[30] Humphries RM, Ambler J, Mitchell SL, Castanheira M, Dingle T (2018). CLSI methods development and standardization working group best practices for evaluation of antimicrobial susceptibility tests. J Clinical Microbiol.

[31] Sakarikou C, Altieri A, Bossa MC, Minelli S, Dolfa C (2018). Rapid and cost-effective identification and antimicrobial susceptibility testing in patients with Gram-negative bacteremia directly from blood-culture fluid. J Microbiol Methods.

[32] Kok J, Thomas LC, Olma T, Chen SCA, Iredell JR (2011). Identification of bacteria in blood culture broths using matrix-assisted laser desorption-ionization Sepsityper and time of flight mass spectrometry. PLoS One.

[33] Barnini S, Brucculeri V, Morici P, Ghelardi E, Florio W (2016). A new rapid method for direct antimicrobial susceptibility testing of bacteria from positive blood cultures. BMC Microbiol.

[34] Chen JHK, Ho P-L, Kwan GSW, She KKK, Siu GKH (2013). Direct bacterial identification in positive blood cultures by use of two commercial matrix-assisted laser desorption ionization-time of flight mass spectrometry systems. J Clin Microbiol.

[35] Falconer K, Hammond R, Parcell BJ, Gillespie SH (2024). Rapid determination of antimicrobial susceptibility of Gram-negative bacteria from clinical blood cultures using a scattered light-integrated collection device. J Med Microbiol.

[36] Van den Poel B, Meersseman P, Debaveye Y, Klak A, Verhaegen J (2020). Performance and potential clinical impact of Alfred60^AST^ (Alifax®) for direct antimicrobial susceptibility testing on positive blood culture bottles. Eur J Clin Microbiol Infect Dis.

[37] Desmet S, Verhaegen J, Glupzcynski Y, Van Eldere J, Melin P (2016). Development of a national EUCAST challenge panel for antimicrobial susceptibility testing. Clin Microbiol Infect.

[38] Pancholi P, Carroll KC, Buchan BW, Chan RC, Dhiman N (2018). Multicenter evaluation of the accelerate phenotest BC kit for rapid identification and phenotypic antimicrobial susceptibility testing using morphokinetic cellular analysis. J Clin Microbiol.

[39] Pantel A, Monier J, Lavigne JP (2018). Performance of the accelerate pheno system for identification and antimicrobial susceptibility testing of a panel of multidrug-resistant Gram-negative bacilli directly from positive blood cultures. J Antimicrob Chemother.

[40] Charnot-Katsikas A, Tesic V, Love N, Hill B, Bethel C (2018). Use of the accelerate pheno system for identification and antimicrobial susceptibility testing of pathogens in positive blood cultures and impact on time to results and workflow. J Clin Microbiol.

[41] Descours G, Desmurs L, Hoang TLT, Ibranosyan M, Baume M (2018). Evaluation of the accelerate pheno system for rapid identification and antimicrobial susceptibility testing of Gram-negative bacteria in bloodstream infections. Eur J Clin Microbiol Infect Dis.

[42] Schneider JG, Wood JB, Schmitt BH, Emery CL, Davis TE (2019). Susceptibility provision enhances effective de-escalation (SPEED): utilizing rapid phenotypic susceptibility testing in Gram-negative bloodstream infections and its potential clinical impact. J Antimicrob Chemother.

[43] Cenci E, Paggi R, Socio GVD, Bozza S, Camilloni B (2020). Accelerate pheno blood culture detection system: a literature review. Future Microbiol.

[44] Burnham JP, Wallace MA, Fuller BM, Shupe A, Burnham C-AD (2019). Clinical effect of expedited pathogen identification and susceptibility testing for Gram-negative bacteremia and candidemia by use of the accelerate pheno system. J Appl Lab Med.

[45] Banerjee R, Komarow L, Virk A, Rajapakse N, Schuetz AN (2021). Randomized trial evaluating clinical impact of RAPid IDentification and susceptibility testing for Gram-negative bacteremia: RAPIDS-GN. Clin Infect Dis.

[46] Giordano C, Piccoli E, Brucculeri V, Barnini S (2018). A prospective evaluation of two rapid phenotypical antimicrobial susceptibility technologies for the diagnostic Stewardship of sepsis. Biomed Res Int.

[47] Elliott G, Malczynski M, Barr VO, Aljefri D, Martin D (2019). Evaluation of the impact of the accelerate pheno system on time to result for differing antimicrobial Stewardship intervention models in patients with Gram-negative bloodstream infections. BMC Infect Dis.

[48] De Socio GV, Belati A, Paggi R, D’Arpino A, Moretti A (2020). Accelerate pheno system in sepsis by Gram-negative pathogens: four months of hospital experience. New Microbiol.

[49] Marschal M, Bachmaier J, Autenrieth I, Oberhettinger P, Willmann M (2017). Evaluation of the accelerate pheno system for fast identification and antimicrobial susceptibility testing from positive blood cultures in bloodstream infections caused by Gram-negative pathogens. J Clin Microbiol.

[50] Anton-Vazquez V, Adjepong S, Suarez C, Planche T (2019). Evaluation of a new rapid antimicrobial susceptibility system for Gram-negative and Gram-positive bloodstream infections: speed and accuracy of Alfred 60AST. BMC Microbiol.

[51] Mantzana P, Netsika F, Arhonti M, Meletis G, Kandilioti E (2021). Performance evaluation of Alfred^60^AST rapid susceptibility testing directly from positive blood cultures in the routine laboratory workflow. Eur J Clin Microbiol Infect Dis.

[52] Cupaiolo R, Cherkaoui S, Serrano G, Dauby N, Georgala A (2022). Antimicrobial susceptibility testing determined by Alfred 60/AST (Alifax®) in a multi-sites lab: performance’s evaluation and optimization of workflow. J Microbiol Methods.

[53] Kim J-H, Kim TS, Jung HG, Kang CK, Jun K-I (2019). Prospective evaluation of a rapid antimicrobial susceptibility test (QMAC-dRAST) for selecting optimal targeted antibiotics in positive blood culture. J Antimicrob Chemother.

[54] Choi J, Yoo J, Lee M, Kim E-G, Lee JS (2014). A rapid antimicrobial susceptibility test based on single-cell morphological analysis. Sci Transl Med.

[55] Anton-Vazquez V, Suarez C, Planche T (2021). Impact of rapid susceptibility testing on antimicrobial therapy and clinical outcomes in gram-negative bloodstream infections. J Antimicrob Chemother.

[56] Bouza E, Sousa D, Muñoz P, Rodríguez-Créixems M, Fron C (2004). Bloodstream infections: a trial of the impact of different methods of reporting positive blood culture results. *Clin Infect Dis*.

[57] Garnacho-Montero J, Garcia-Garmendia JL, Barrero-Almodovar A, Jimenez-Jimenez FJ, Perez-Paredes C (2003). Impact of adequate empirical antibiotic therapy on the outcome of patients admitted to the intensive care unit with sepsis. Crit Care Med.

[58] Kerremans JJ, Verboom P, Stijnen T, Hakkaart-van Roijen L, Goessens W (2008). Rapid identification and antimicrobial susceptibility testing reduce antibiotic use and accelerate pathogen-directed antibiotic use. J Antimicrob Chemother.

[59] Ling TK, Tam PC, Liu ZK, Cheng AF (2001). Evaluation of VITEK 2 rapid identification and susceptibility testing system against gram-negative clinical isolates. J Clin Microbiol.

